# *Notes from the Field*: Molecular Detections of *Cladophialophora bantiana* at a Clinical Reference Laboratory — United States, 2009–2026

**DOI:** 10.15585/mmwr.mm7540e1

**Published:** 2026-10-15

**Authors:** Maria T. Nelson, Thao T. Truong, Lori A. Bourassa, Dallas J. Smith, Joshua A. Lieberman

**Affiliations:** ^1^Department of Laboratory Medicine and Pathology, University of Washington School of Medicine, Seattle, Washington; ^2^Mycotic Diseases Branch, Division of Foodborne, Waterborne, and Environmental Diseases, CDC.

**Summary**

**What is already known about this topic?**

*Cladophialophora bantiana* is a neurotropic fungus; infection is associated with a 60%–70% case-fatality rate. National surveillance does not exist, and neither the environmental reservoir nor the route of infection is fully understood.

**What is added by this report?**

During January 2009–July 2026, 48 confirmed cases and one probable case of *C. bantiana* were detected in clinical samples (predominantly central nervous system tissue [88%]) originating in 22 jurisdictions and tested at a single clinical reference laboratory; detections of the fungus increased approximately fourfold from 2023 to 2025.

**What are the implications for public health practice?**

Reasons for the apparent increase are unknown. Further research into epidemiologic risk factors and potentially evolving virulence are warranted. Systematic reporting of *C. bantiana* cases is needed and *C. bantiana* should be considered in patients with a brain abscess. Patients with infection require prompt antifungal therapy, and surgical excision might improve patient outcomes.

During 2017–2023, the number of cases of *Cladophialophora bantiana* detected by a clinical reference laboratory at the University of Washington (UW) increased substantially. *C. bantiana* is a highly virulent, neurotropic dematiaceous mold*; infection is associated with a 60%–70% case-fatality rate, even with aggressive therapy (*1*). Approximately one half of patients with *C. bantiana* infections have no preceding immunocompromising condition (*1*). Despite its virulence, relatively little is known about the risk factors for or epidemiology of this fungus. The presumed route of infection is inhalation or direct inoculation after cutaneous injury, followed by hematogenous spread to the brain. Rarely, pulmonary (*2*) and cutaneous (*3*) infections have been reported; most cases occur as brain abscesses, often with no extracranial disease and no clear source of inoculation. Furthermore, *C. bantiana* has only rarely been isolated from environmental sources, and many patients do not have similar exposure histories or risk factors (*1*). No routine surveillance is available; data consist primarily of case reports and retrospective literature reviews, limiting assessment of evolving epidemiologic characteristics and patterns. This report describes a substantial increase in the rate of detection of *C. bantiana* at a molecular microbiology clinical reference laboratory, with cases originating from a wide geographic range in the United States.

## Investigations and Outcomes

### Methods

All cases of *C. bantiana* detected by broad-range fungal polymerase chain reaction (PCR)^†^ at the UW laboratory during January 2009–July 2026 were compiled. Cases were categorized by test order year, tissue site, and state of origin, determined both by referring laboratory location and surgical pathology report, when available.^§^ To control for increased testing over time, the number of *C. bantiana* cases was normalized to that of all intracranial central nervous system (CNS) specimens submitted for broad-range fungal PCR during the same period. The DNA extraction method was changed in February 2021; the remainder of the assay has not changed substantially over time. Data were analyzed in R (version 4.5.2; R Foundation). This activity was approved by the institutional review boards at UW and was reviewed by CDC, deemed not research, and conducted consistent with applicable federal law and CDC policy.^¶^

### Characteristics of Detected *C. bantiana* Cases

During January 2009–July 1, 2026, a total of 48 confirmed cases of *C. bantiana* infection and one probable case** were identified by broad-range fungal PCR; the median patient age was 68 years (IQR = 63–74 years). Among these 49 cases, 43 (88%) were detected in intracranial CNS tissue specimens and six (12%) in lung specimens. No extracranial CNS infections were identified. During 2009–2023, the number of cases per year ranged from one to five. Case counts increased to eight in 2024 and 14 in 2025 (Figure). During 2017–2023, an average of 8.4 cases of *C. bantiana* were detected per 1,000 CNS specimens submitted for broad-range fungal PCR at UW. By 2025, this increased by 210%, to 26 per 1,000, suggesting that the increase in *C. bantiana* cases is not attributable to an increase in laboratory referral volume alone. Cases originated from 21 U.S. states and the District of Columbia (Supplementary Figure); the largest number of cases originated from California (six), Florida (five), Texas (five), and Pennsylvania (four); state of origin was not available for three cases. Broad-range fungal PCR testing of cerebrospinal fluid was performed antemortem for the single, fatal case managed at the UW Medical Center and was negative for *C. bantiana* in samples collected concurrently and 1 month before biopsy. Outcome information for other cases was not available.

**FIGURE:**
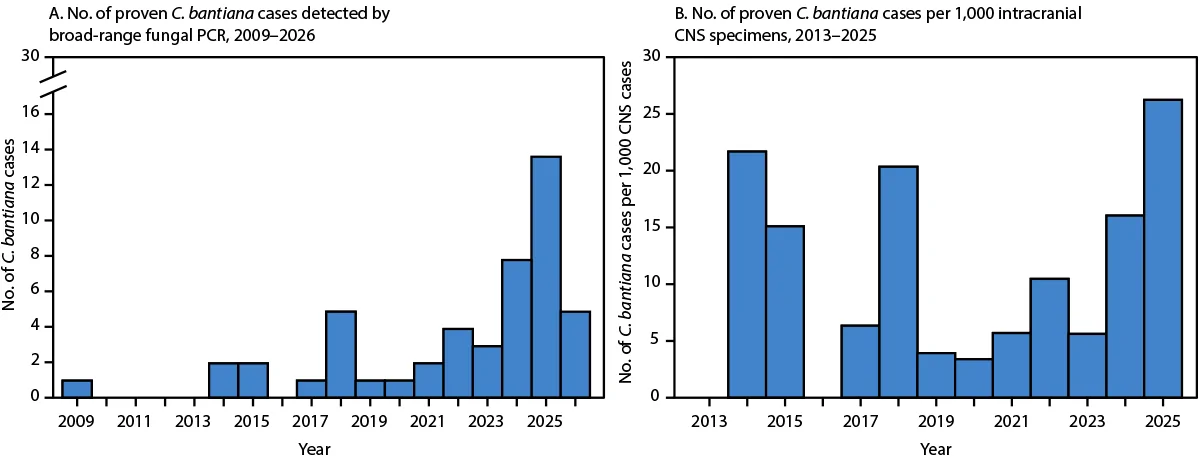
Number of proven *Cladophialophora bantiana* cases detected by broad-range fungal polymerase chain reaction (A)* and number of *C. bantiana* cases per 1,000 intracranial central nervous system specimens submitted for the same assay (B),^†^ by year — United States, January 2009–July 2026 **Abbreviations:** CNS = central nervous system; PCR = polymerase chain reaction. * Data for 2026 are through July 1. ^†^ Baseline CNS cases were available beginning in 2013.

## Preliminary Conclusions and Actions

*C. bantiana* is a highly virulent mold that predominantly causes brain abscesses in both immunocompetent and immunocompromised persons. The frequency of *C. bantiana* cases detected by broad-range fungal PCR at UW per 1,000 total submitted CNS specimens increased approximately fourfold in 2 years (2023–2025). Whereas most cases were detected in CNS specimens, detection of 12% of cases in pulmonary samples supports the presumed respiratory route of infection. Because of the nature of reference laboratory testing, limited demographic, occupational, and clinical data were available, and the causes of this recent increase in cases are unclear, highlighting the need for more systematic reporting mechanisms for this mold and for clinical laboratories to publish numbers of detected cases. This approach could help in collecting epidemiologic data to identify risk factors and transmission patterns more effectively. Clinicians should be aware of this increase in incidence and consider *C. bantiana* in patients who have signs and symptoms of a brain abscess. Testing can be ordered through clinical reference laboratories that offer molecular testing for fungi in tissue or CSF specimens. In a 2024 study of 23 patients in France (*4*), surgical excision or drainage of brain abscesses was associated with more favorable outcomes; thus, when possible, surgical exision should be considered, concurrently with administration of combination antifungal therapy. Although the most effective antifungal regimen is not known, triple therapy (treatment with lipsosomal amphotericin B, posaconazole or voriconazole, and flucytosine) for at least 2 weeks has demonstrated some effectiveness (*4*).

## Footnotes

* A large, diverse group of molds and black yeasts defined by the presence of melanin or melanin-like pigments in their cell walls.

† This fungal organism–agnostic method amplifies conserved ribosomal RNA loci (28S D1/D2, ITS1, and ITS2) directly from fresh and formalin-fixed paraffin-embedded tissue samples or body fluids and performs sequencing to assign taxonomic identification of detected fungal DNA.

§ State of origin was unavailable for three cases.

¶ 45 C.F.R. part 46, 21 C.F.R. part 56; 42 U.S.C. Sect. 241(d); 5 U.S.C. Sect. 552a; 44 U.S.C. Sect. 3501 et seq.

** One case of intracranial CNS infection was reported in 2024 as a *Cladophialophora* species infection with a significant sequence homology to *C. bantiana.*
